# Pathomechanisms of Vascular Depression in Older Adults

**DOI:** 10.3390/ijms23010308

**Published:** 2021-12-28

**Authors:** Kurt A. Jellinger

**Affiliations:** Institute of Clinical Neurobiology, Alberichgasse 5/13, 1150 Vienna, Austria; jellinger@univie.ac.at; Tel./Fax: +43-1-526-6534

**Keywords:** late-life depression, vascular depression, cerebral small vessel disease, microvascular dysfunction, white matter lesions, metabolic factors, management problems

## Abstract

Depression in older individuals is a common complex mood disorder with high comorbidity of both psychiatric and physical diseases, associated with high disability, cognitive decline, and increased mortality The factors predicting the risk of late-life depression (LLD) are incompletely understood. The reciprocal relationship of depressive disorder and age- and disease-related processes has generated pathogenic hypotheses and provided various treatment options. The heterogeneity of depression complicates research into the underlying pathogenic cascade, and factors involved in LLD considerably differ from those involved in early life depression. Evidence suggests that a variety of vascular mechanisms, in particular cerebral small vessel disease, generalized microvascular, and endothelial dysfunction, as well as metabolic risk factors, including diabetes, and inflammation that may induce subcortical white and gray matter lesions by compromising fronto–limbic and other important neuronal networks, may contribute to the development of LLD. The “vascular depression” hypothesis postulates that cerebrovascular disease or vascular risk factors can predispose, precipitate, and perpetuate geriatric depression syndromes, based on their comorbidity with cerebrovascular lesions and the frequent development of depression after stroke. Vascular burden is associated with cognitive deficits and a specific form of LLD, vascular depression, which is marked by decreased white matter integrity, executive dysfunction, functional disability, and poorer response to antidepressive therapy than major depressive disorder without vascular risk factors. Other pathogenic factors of LLD, such as neurodegeneration or neuroimmune regulatory dysmechanisms, are briefly discussed. Treatment planning should consider a modest response of LLD to antidepressants, while vascular and metabolic factors may provide promising targets for its successful prevention and treatment. However, their effectiveness needs further investigation, and intervention studies are needed to assess which interventions are appropriate and effective in clinical practice.

## 1. Introduction

The pathophysiology of late-life depression (LLD) is multifactorial and complex. This common complex mood disorder involves individuals where the initial depressive episode occurs after age 60–65 in the absence of a previous history of affective illness [1,2]. Major depression and depressive episodes in older adults that may induce cognitive and executive deficits [3,4] are associated with lower quality of life [5,6], high comorbidity of both other psychiatric and physical diseases, and increased mortality risk [7,8,9,10,11,12]. LLD also poses a great socioeconomic burden by increasing costs on health care and on the workplace due to functional impairment. The prevalence of major depression disorder (MDD) varies largely and ranges from 0.9% to 42% among older adults; clinically relevant depressive symptoms are present in 7.2% to 49% [13], and in 32% of patients with mild cognitive impairment (MCI) [14]. Worldwide the prevalence of LLD is increasing, particularly in lower-income countries, which reflects both the overall growth and aging of the global population [15]. In Korean elders, vascular depression (VaD) was reported to make up about half of all MDD cases [16]. Older age appears to be a consistent and important risk factor for a poorer, more persistent course of depression [17], and elderly individuals usually show a chronic course of depressive symptoms. Among individuals aged above 60 years with depression, 61% reported a persistent, chronic course of depressive symptoms [18]. Accordingly, the etiology and pathogenesis of LLD may differ from that involved in early life. The last decades have witnessed considerable advances in our understanding of the neurobiology of early- and late-onset depression and have shown that disturbances of fronto–subcortical functioning due to cerebrovascular and other brain disorders are implicated in LLD. However, its etiopathogenetic features and the molecular basis are still poorly understood [19,20].

It is important to distinguish between LLD with early- and late-onset [21,22], for review see [12]. Early-onset depression may predispose to LLD by accumulating depressive episodes over a lifetime and is associated with a positive family history of mood disorders, lower cardiovascular risk factors, and lower risk for cognitive decline, while late-onset depression (LOD) shows a higher risk of cardiovascular risk burden, a higher risk for cognitive decline, and greater treatment resistance [23]. These overt differences generated mechanistic hypotheses on the role of vascular and metabolic risk factors and their involvement in the development of LLD [24]. It was postulated that cerebrovascular damage may contribute to depression in advanced age via involvement of brain regions involved in mood regulation, notably damage to subcortical regions [25]. “Vascular depression” or “subcortical ischemic depression”, due to lesions in the subcortical white and/or gray matter caused by reduced cerebral blood flow (CBF), were suggested to be particularly relevant in older individuals without a history of previous depressive episodes, i.e., LOD. VaD is regarded as a subtype of LLD, characterized by a distinct clinical presentation associated with cerebrovascular or related brain damage. VaD encompasses not only depression with cerebral small vessel disease but also post-stroke depression (PSD) and depression related to myocardial infarction and other cardiovascular diseases [26]. Although depressive symptoms in older adults (around/after age 60 years) are common, about 22% of adults with lifetime MDD meet the criteria for the VaD subtype [27], the concept of age-related VaD is still not generally accepted and it is not included in current psychiatric manuals as DSM-5 and ICD-10 [25]. The Diagnostic and Statistical Manual of Mental Disorders (DSM-V) defines major depressive disorder by the presence of depressed mood or a marked loss of interest or pleasure in activities along with five or more of the following associated symptoms: changes in appetite or weight (5% of the total body weight), sleep, energy, concentration, and psychomotor activity, feelings of inappropriate guilt or worthlessness, and recurrent thoughts of death or suicide. There should be an impairment in social, occupational, and other areas of functioning. LLD is underdiagnosed and inadequately treated [2].

## 2. The “Vascular Depression Hypothesis”

**Figure 1:**
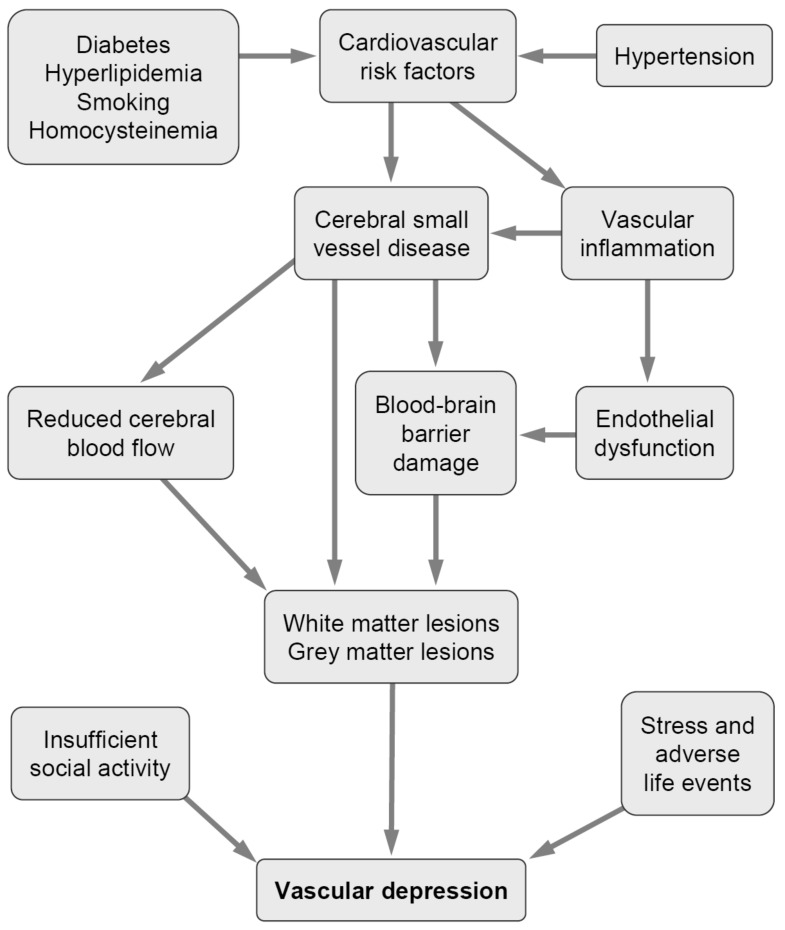
Flow chart of possible mechanisms of vascular depression.

Introduced by Alexopoulos et al. [28], it proposed that cerebral small vessel disease (CSVD), in particular subcortical microvascular dysfunction and white matter hyperintensities (WMH), microbleeds and lacunes in gray matter, may predispose, precipitate, and perpetuate depressive symptoms in aged people as a consequence of structural damage in the fronto–subcortical circuits [24,28,29]. This hypothesis is based on the observation that cerebrovascular risk factors are frequently present in aged individuals with depression, its comorbidity with cerebrovascular lesions, and the frequent development of depression after stroke, affecting between 20% and 30% of all stroke patients [30,31,32], for review see [12]. Cerebrovascular burden in midlife predicts depressive symptomatology in later life, suggesting an interrelation between cerebrovascular burden and LLD [33]. A clinical definition regards cerebrovascular risk factors as one of the cardinal factors of VaD [34] (Figure 1). Moreover, such a vascular etiology may explain the high recurrence and persistence rate of LLD, in addition to high treatment resistance to antidepressant and/or behavioral therapy [12,35,36,37]. Later, the term “MRI-defined VaD” was introduced [38], with a focus on the presence and severity of either white matter changes or subcortical gray matter lesions (lacunes, microbleeds), supporting the hypothesis that loss of brain volume, diminished white matter integrity, and lesions in the fronto–striatal–limbic regions are associated with depressive symptoms in the elderly [39,40,41,42], although this was not confirmed by others [43,44,45,46,47].

## 3. Clinical Features of Vascular Depression

The clinical presentation of VaD is characterized by psychomotor slowing, lack of initiative, anhedonia, apathy, executive dysfunction, pronounced disability, impaired processing speed, risk of cognitive impairment, lack of insight and suspiciousness, but less prominent depressive ideation, a mild vegetative syndrome, and poorer outcome summarized as “depressive–executive syndrome” [48]. Other clinical factors distinguish VaD from non-vascular depression in the elderly, including age, higher cardiac illness burden, and greater deficits in self-initiation and concentration, without risk of suicidal activity, agitation, a negative family history of depression, and negative history of previous depressive episodes [49,50] (see Table 1). Patients with vascular dysfunction and depression exhibit significantly higher aggressive and autoaggressive tendency due to a lower level of tolerance threshold [51], while others found no evidence to support the hypothesis that depressed older persons with vascular disease have a distinct depression profile, but they showed more disability than those without vascular disease [52]. A large community study failed to identify a difference in the depression symptom profile in incident cardiac and non-cardiac cases at follow-up [53]. While loss of energy, lethargy, apathy, and executive dysfunction are frequently observed in patients with VaD, other symptoms, namely psychomotor retardation and anhedonia, were not significantly associated with vascular risk factors [54], and no significant correlations between vascular risk factors and depressive symptoms were found in MRI-defined subcortical ischemic depression [55]. A recent Consensus Report suggested the following criteria for VaD [23]: (1) evidence of vascular pathology in the elderly with or without cognitive impairment [56]; (2) absence of previous depressive episodes preceding obvious CVD; (3) presence of cerebrovascular risk factors; (4) co-incidence of depression with cerebrovascular risk factors; (5) clinical symptoms characteristic of VaD (executive dysfunction, decrease in processing speed, lethargy, etc.), and (6) neuroimaging data confirming CVD. However, the multifold pathogenesis of VaD as a possible subtype of LLD needs further elucidation.

## 4. VaD and Cognitive Impairment

Individuals with VaD are at greater risk of developing cognitive impairment (CI), which is particularly high in individuals with depression and vascular disorder [3]. This combination is highly prevalent among elderly subjects with MCI [14] and in cognitively elders with an increased risk of developing MCI, while depression was associated with severity of subcortical WMH [57]. Neuroradiological evidence of CSVD is a significant marker for cognitive deficits [58,59]. Although hemodynamic dysfunction may play a pathogenic role in the development of CI in patients with VaD [60], a number of other pathogenic factors, such as disorders of the glutamatergic system [20], stress-induced neurovascular pathology [61], mitochondrial dysfunction, lipid dysmetabolism [62,63], neuroimmune regulatory mechanisms [64,65,66], excitotoxicity, chronic inflammation [67], deficits of nerve growth factors [68], and others, may contribute to cognitive deficits in the presence of cerebrovascular burden, which is common in VaD [23,34]. Vascular risk factors, especially hypertension and diabetes, are higher in patients with LLD and affect the severity of depression and the degree of cognitive impairment [56]. Thus, the brains of older adults with LLD may be structurally and functionally more compromised beyond just CSVD, rendering those with depression more likely to express cognitive deficits [69]. Consistent with this view, VaD is associated with alterations of functional connectivity of the default mode and cognitive control networks [70]. Diffusion tensor imaging (DTI) data from persons with depression and MCI showed a significant decrease in regional controllability of the left/right superior prefrontal cortex and cingulate gyrus [71]. Fundamental questions remain regarding the relationship between depression and cognitive manifestations in VaD, particularly in individuals without overt neurocognitive syndrome. One possibility is that the neurobiological processes that predispose elderly individuals to LLD may exacerbate cognitive vulnerabilities in the presence of CVD, which may contribute to the cognitive heterogeneity associated with white matter abnormalities, thus accentuating impairments in higher-order cognitive domains. This is supported by studies demonstrating WMHs and lacunes of presumed vascular origin in patients with LLD which predicted poorer performance on delayed memory and executive function [72,73,74,75,76,77]. Dispersion as a marker of neurocognitive integrity was correlated with white matter lesions in LLD but not in healthy controls [78]. Since cognitive dysfunction may precede depression and persist despite symptomatic remission [79], LLD becomes challenging to distinguish it from dementia, since both have overlapping symptomatic profiles, especially when depression affects the cognition and presents as “pseudodementia” [71].

## 5. Cerebral Small Vessel Disease

Accumulating evidence suggests that CSVD may contribute to the onset and progression of VaD by inducing chronic ischemia in brain tissue [80]. MRI stigmata of CSVD (WMH, lacunes, microbleeds, enlarged perivascular spaces, and cerebral atrophy) are frequently associated with depression and incident stroke [81], and markers of progression of CSVD over time were associated with a higher incidence of depressive symptoms in a general elderly population [82]. Patients with CADASIL exhibited frequencies of 17.9% for MDD, which increased to 46.2% as WMH volume increased, while the effects of lacunar infarctions and cortical microbleeds were not significant [83]. A systemic review and meta-analysis investigated the association of several markers of CSVD with both prevalent and incident LLD, showing a longitudinal association between higher WMH volume and incident LLD [84]. However, the individual studies included in this meta-analysis showed mixed results, probably due to suboptimal assessment of WMHs (rating scales vs. semi-automated volumetry) or depression (self-reported vs. clinical diagnosis), or in the wide variation in age of included populations. As CSVD is more common among the elderly, its relevance for depression becomes larger with advancing age. In agreement with this hypothesis, a recent population-based study on the longitudinal association of markers of CSVD and brain atrophy on the incidence and course of depressive symptoms in elderly individuals revealed that large WMH volume due to CSVD is associated with chronic depression above the age of 60 years. WMH volume possibly provides a preventive target for LLD, while general brain atrophy markers were not associated with incident or chronic depression [22], for a recent review of CSVD in LLD also see [12]. Moreover, no significant association of these CSVD markers and depression was found for individuals below the age of 60 years, while others also underscored the association of LLD with increased WMH burden, which is in line with the VDH [72]. Another recent study combined the presence of lacunar infarcts, cerebral microbleeds, and WMHs in a combined CSVD score, but did not find an association between this and incident depression [21]. Moreover, CSVD was associated with apathy in the general population, supporting the hypothesis of vascular apathy [85]. Individuals with VaD and CSVD show alterations of functional connectivity of the default mode and cognitive control networks [70] and may also show a systemic impact on structural brain networks [86]. Greater CSVD burden was associated with greater Geriatric Depression Scale (GDS), WMH, and subcortical lacunes, but not with amyloid deposition or cortical thinning, the network changes mediating the relationship of WMH and lacunes, both consequences of CSVD, with GDD [74]. In conclusion, there is convincing evidence on the role of WMHs in the development and progression of LLD, supporting the pathogenic contribution of CSVD in the development of VaD. Thus, LLD appears to be a prodrome or accelerating factor for cognitive deterioration.

## 6. Impaired Cerebral Hemodynamics

There is recent epidemiological evidence that microvascular dysfunction (MVD) could be a generalized process underlying the development of many chronic diseases including LLD [84], and that LLD may be a manifestation of a larger phenotype of cerebral and general MVD [87]. MVD is defined as dysfunction of the microcirculation that consists of blood vessels with a diameter 150µm, including arterioles, capillaries, venules, and some specialized structures, such as arteriovenous shunts [88]. Microcirculation delivers oxygen and nutrients to and removes the waste from the tissue [89]. Furthermore, it plays an important role in the autoregulation of the blood pressure, especially in cerebral autoregulation, in which the blood pressure remains constant despite large variabilities in the periphery [90], for review see [12]. Reduced blood flow velocity studied by transcranial Doppler ultrasonography of large cerebral vessels, which may indicate cerebral microangiopathy, predicted depressive disorders in healthy older adults [91]. Modern technologies enable the non-invasive assessment of MVD in different organs, including the brain [92,93]. This may be an alternative for measuring MVD to the more expensive brain MR imaging. Whether MVD is a generalized phenomenon throughout the body and the brain remains to be elucidated, but both markers of generalized MVD and CSVD have been associated with LLD [84]. Extensive studies of cerebral hemodynamics in LLD patients revealed that they were more prone to intracranial arterial stenosis. More severe stenosis of the middle cerebral artery showed significantly more severe hypoperfusion of the frontal and parietal lobes and significantly higher WMH loads. This could be used as an imaging biomarker to indicate diffuse or localized cerebral macro- and microvascular pathology in LLD [94]. Perfusion changes in deep nuclei and cerebellum reflect abnormal activities in LLD patients but suggest that microvascular pathology affects neurovascular coupling in ventral circuits. It is suggested that microvascular pathology is important in the etiology of VaD as well as for interferences about resulting brain activity changes [95]. Endothelin dysfunction could contribute to the occurrence of VaD due to an association between risk factors for endothelial damage (i.e., hyperglycemia, hypertension, dyslipidemia) and depression [96]. The vascular endothelium is a single cellular layer that lines the walls of all blood vessels. Releasing both vasodilators (i.e., nitric oxide) and vasoconstrictors (i.e., endothelin), it regulates the vasomotor tone, thus exerting a fundamental role in the prevention of cerebro–cardiovascular disorders [97]. Biomarkers of endothelial dysfunction as measured in plasma have been related to LLD in cross-sectional studies [98,99]. A study assessing the association of markers of MVD with the incidence of LLD found plasma markers of endothelial dysfunction to be associated with a 1.19-fold increased risk for LLD [100]. Such biomarkers of endothelial dysfunction include endothelin-1 (ET-1), circulating endothelial cells, endothelial microparticles, endoglin, etc. [101], plasma soluble intercellular adhesion molecules (siCAM), or high-sensitive C-reactive particles (hs-CRP) [102]. A genome-wide association study (GWAS) suggested a potential role of vascular endothelial growth factor (VEGF) in depression development [103]. VEGF levels were significantly elevated in individuals with MDD as compared to healthy controls, which indicated that VEGF may be a biomarker for VaD [104]. Furthermore, endothelial dysfunction was associated with a persistent course of depression [105]. More recent evidence for the role of systemic damage of the endothelium was found in a study showing that markers for endothelial dysfunction were associated with reduced CBF in LLD patients [102,106]. In conclusion, these studies suggest that macro- and microvascular dysfunctions causing impaired cerebral hemodynamics/reduced perfusion play a role in the development of LLD.

## 7. Metabolic Risk Factors

Not only CSVD but also upstream vascular and metabolic risk factors, such as hypertension, dyslipidemia, hyperglycemia (type 2 diabetes), and obesity, may be involved in the etiology of LLD [34]. The metabolic syndrome, a combination of all these factors in one term [107], estimated to affect over a billion people worldwide [108], is a risk factor for WMHs and lacunar infarcts [109], which may explain its probable association with LLD. Moreover, the prevalence of depression is nearly doubled in individuals with type 2 diabetes as compared with the general population [110], and the association between duration of diabetes and risk of current depression was “J-shaped” with odds ratios of 1.92 to 3.13 for under 10 and over 30 years of diabetes [111]. Hyperglycemia, a key feature of type 2 diabetes, may be an important factor involved in the etiology of LLD and cognitive impairment [112,113]. Another possibility explaining the association between hyperglycemia and LLD is its involvement via generalized MVD [80] and inflammation [114], which both may lead to CSVD [115], and subsequent depression [80,116]. Several meta-analyses have examined the role of hyperglycemia in the etiology of LLD and found evidence that continuous hyperglycemia levels may be involved in the development of depression [117], which is in line with a large cross-sectional study demonstrating the association between diabetes and higher prevalence of depression [118]. For a more comprehensive review of metabolic risk factors of LLD see [12]. In conclusion, there is evidence for many metabolic factors, including hyperglycemia, obesity, and type 2 diabetes as important metabolic risk factors in LLD.

## 8. (Neuro)inflammation

There is convincing evidence for a longitudinal association of low-grade inflammation and depression [119], which may become stronger with increasing age due to more chronic neuroinflammation [67,120]. Chronic stress and inflammation combine to compromise vascular and brain function. The resulting increase in proinflammatory cytokines and microglial activation drive brain pathology leading to depression and MCI via blood-brain barrier (BBB) permeability and cytokine production [64]. BBB-associated tight junction disruption has been implicated in the pathophysiology of depression, but the underlying mechanisms and importance remain largely unknown [121]. Recent data indicate that primary microglial activation may result from chronic stress on vascular function and that there is a dynamic crossover between neuroinflammation and other relevant neurobiological correlates of depression [67]. Numerous studies found elevated peripheral and central inflammatory cytokines and acute-phase proteins in depressed individuals and the newest diagnostic criteria for MDD identify inflammation as a possible cause [122]. Pro-inflammatory cytokines may lead to depression via different pathways [123]: the neuronal pathway, in which cytokines activate the glossopharyngeal nerve as a novel pathway in immune-to-brain communication [124], the humoral pathway, in which cytokine signals enter the brain via fluid diffusion [125]. Pro-inflammatory cytokines are transported through the BBB into the brain [126], or activation of IL-1 receptors, located on endothelial cells of cerebral veins, by circulating cytokines results in the local production of prostaglandins [127]. Engagement of these immune regulatory mechanisms ultimately leads to the production of pro-inflammatory cytokines by activated microglial cells in the brain, which can induce symptoms of depression [123]. For a recent review of inflammation in LLD see [12]. Inflammation-induced elevation of cortisol levels is also known to be associated with depression [128]. Moreover, neuroinflammation has been shown to occur in response to acute ischemic stroke and may underlie secondary poststroke pathologies, such as depression, that often develop over time poststroke. The increased production of proinflammatory cytokines, such as IL-1β, TNF-α, or IL-18, resulting from stroke may lead to an amplification of the inflammatory process, particularly in limbic areas, and widespread activation of indoleamine 2,3-dioxygenase and subsequently to depletion of serotonin in paralimbic regions. The resultant dysfunction may lead to PSD [129]. A pilot [^11^C]PBR28 PET/MRI study in chronic stroke patients demonstrated extensive microglial activation in regions outside the infarct zone, possibly related to stroke risk factors, such as hypertension [130]. Furthermore, an association between vascular inflammation and depressive disorder has been observed [106], and there is further evidence for a close relationship between neuroinflammation and depression [67]. Recent studies, however, suggest that there may not be a shared genetic mechanism contributing to MDD and higher inflammation levels [131]. On the other hand, depression represents an independent factor for (cardio-) vascular pathology via inflammation-vascular-autonomic dysregulations. Depression-linked pro-inflammatory reactions are closely associated with endothelial dysfunction and promote arteriosclerosis, resulting in an increased risk of developing CVD in major depression [132]. In addition, an imbalance of the autonomous nervous system—sympathetic overactivity and vagal underactivity—may stimulate pathogenic pathways, leading to the acceleration of atherosclerotic changes and CVD in patients with MDD [133]. In conclusion, there is increasing evidence for an involvement of vascular/neuroinflammation and other low-grade inflammation processes in the pathogenesis of LLD, which also may promote vascular pathology via inflammation-vascular-autonomic dysregulation. Further studies should clarify the pathomechanism linking depression and vascular pathology and vice versa.

## 9. Neurodegeneration

Depression is common in neurodegenerative diseases, such as Alzheimer’s disease (AD), Parkinson’s disease (PD), fronto–temporal lobe degeneration and others [134], Lewy body disease, and others, but LLD can also be an indicator of latent neurodegeneration [135]. Depressive symptoms may develop in the early stages of neurodegeneration, occurring without clear presence of cognitive decline or only mild cognitive deterioration, but they can appear also lately when the neurodegenerative disease is fully developed. In cross-sectional studies, LLD has been associated with brain atrophy, particularly in the hippocampus and orbitofrontal cortex [136]. Lower brain volume, temporal lobe atrophy, and smaller corpus callosum have been related to the incidence of LLD [137,138], but brain atrophy may also be a consequence of depression, and LLD was associated with right frontal atrophy [139]. On the other hand, in a large autopsy series of older adults, MDD was not associated with smaller brain volumes [140]. MRI images in both AD patients with and without depressive symptoms showed cortical thinning in the left parietal and temporal regions. The negative correlation between cortical thickness and cerebrospinal fluid (CSF) tau was greater in depressed compared to non-depressed AD patients in the precuneus and parahippocampal cortex. These findings suggest that depressive symptoms in AD patients are associated with cortical thinning in temporal and parietal regions and that tau pathology in these areas may contribute to depressive symptoms in AD [141]. On the other hand, recent data show that LLD and VaD are no risk factors for AD [142], although older cognitively impaired individuals with depressive episodes have more underlying AD pathology, in particular ß-amyloid (Aß)deposition [143], leading to the amyloid hypothesis of LLD [144].

Amyloid-PET studies showed increased Aβ deposition in frontal, parietal, temporal, and occipital cortex in patients with LLD relative to comparison subjects despite no differences in age, sex, MMSE score, vascular risk factors, and APOE ε4 genotype [145], as well as in elderly depressed patients with different subtypes of MCI, supporting the hypothesis that these patients exhibit greater Aβ load than those without MCI [146]. Greater Aβ burden in the left parietal cortex in LLD patients was correlated with greater depressive symptoms and poorer visuospatial memory [147]. Subjects with LLD and Aβ deposition showed greater volume reduction in the left middle temporal gyrus and had lower functional connectivity in frontal, cortical, and limbic areas, both regional gray matter loss, and alterations in brain networks reflecting impairments caused by amyloid deposition and depression, facilitating the detection of prodromal AD in elderly persons with depression and cognitive dysfunction [148]. Default mode network (DMN) dissociation may be pivotal in linking cerebral Aβ pathology and LLD, aberrant anterior DMN functional connectivity being correlated with the severity of depression, whereas posterior DMN functional connectivity was negatively correlated with cognitive function. The anterior and posterior DMN FC dissociation pattern may be pivotal in linking cerebral Aβ pathology and LLD in the course of AD progression. [149]. Contrary to expectation, according to recent findings from the AD neuroimaging initiative (ADNI) depression project study, the LLD group showed less Aβ deposition than the nondepressed group, and Aβ deposition was not associated with depression history characteristics. Aβ was associated with worse memory performance, but this relationship did not differ between LLD and non-depressed persons. This suggests that memory deficits and accelerated cognitive decline reported in LLD patients may not be due to greater cortical Aβ deposition [150].

LLD could be a risk factor or a prodromal phase of AD; this has led to investigating the link between depression and Aβ peptides by measuring Aβ levels in plasma, CSF, and brains in elderly depressed subjects. Low plasma Aβ42 profiles are strongly associated with depression severity, while CSF Aβ-42 levels are lower in depressed than in controls. Elderly depressed with cardiovascular disease risk factors have frequently higher plasma Aβ40 levels and drug resistance; those without these comorbidities have low plasma Aβ42 levels and a greater cognitive impairment [151]. On the other hand, an earlier prospective longitudinal study of older adults showed that higher plasma Aβ42 levels at baseline predicted the development of first-episode LLD and conversion to possible AD, but no association was found between plasma Aβ42 levels and Geriatric Depression Scale (GDS). Depression as measured by scores on GDS either alone or as an interaction factor with plasma Aβ42 levels failed to predict conversion to AD at 5 years [152].

Concurrent changes in depression and cognition among older individuals with higher cortical amyloid burden moderated the association between worsening depressive symptoms and declining cognition, suggesting that depressive symptoms may serve as a target in delaying the clinical course of AD [153]. Despite these and other findings indicating an association between increased amyloid burden in specific areas, e.g., precuneus/posterior cingulate cortex, and depressive symptoms, in general, depression in AD is different from that in AD [8], psychotic symptoms being more common in VaD than in AD [154], and several studies failed to identify a relationship between AD pathology and LLD. Very low-level dysphoria, apathy, anhedonia, and other depressive symptoms may point to neurodegeneration in AD-related regions, but this association appeared to be independent of amyloid burden [155]. Lower hippocampal volume was not related to amyloid pathology in patients with LLD, which counters the belief that changes in hippocampal volume in LLD are due to prodromal AD [156]. Neuropathological studies found no significant association between depressive symptoms, cognitive status, neuritic plaques, and neurofibrillary tangles scores and their interactions, and no differences in neuritic pathology or neuronal density between subjects with major depression and non-depressed controls, making unclear whether and what aspects of neuropathological changes of AD are related to LLD or VaD [34]. In a large community sample autopsy study of aged subjects in which LLD was associated with cognitive impairment (*p* 0.001), moderate or frequent neuritic plaque density was not associated with LLD; hence, the link between depression and dementia may be complex and determined by multiple factors [157]. Other pathways connecting LLD and dementia have been reviewed recently [158].

## 10. Structural Brain Lesions in VaD

Structural and functional imaging studies provided information about underlying structural changes on VaD with reference to location, size, and extent of gray and white matter lesions [159]. MRI-defined VaD requires neuroimaging evidence of cerebrovascular changes, in particular, WMHs and subcortical microinfarcts (lacunes), which may predate the development of depressive symptoms. Microstructural brain lesions, especially WMHs, are more frequently found in individuals with LLD compared to age-matched controls [45,94,160]. LLD-associated structural neuroimaging markers, such as WMH, white matter integrity measured by diffusion tensor imaging, cortical and subcortical volumes, and cortical thickness may provide a structural basis for brain network dysfunction in LLD. It is associated with greater severity or volumes of deep, periventricular, or overall WMH, with decreased white matter integrity of the brain regions belonging to the fronto-striatal-limbic circuits, and reduced white matter tract integrity, which connects these circuits, such as the cingulum, uncinate fasciculus and frontal projections to the corpus callosum [77,161,162]. Functional connectivity and behavioral analyses revealed a disruption of ascending mesostriato–cortical reward signals in LLD and a failure of cortical contingency encoding in the elderly with poor executive control [163]. In LLD, WMHs are associated with region-specific disruptions in cortical and subcortical gray matter areas involved in attentional aspects of cognitive control systems and sensorimotoric processing [164]. On the other hand, no statistical differences were found between VaD and non-VaD patients in cortical thickness of the bilateral precuneal, entorhinal, and parahippocampal cortices or hippocampal volume, which raises several diagnostic and etiological possibilities, namely that VaD may not be connected with other late-life psychiatric illnesses, such as MCI or dementia, and that vascular disease may not be a common etiological risk factor for depression and dementia [165]. Regional gray matter volume is linked with white matter integrity of the uncinate fasciculus in the orbitomedial prefrontal limbic network, and its disruption appears to be involved in the pathophysiology of LLD [166]. Decreased volumes of cortical thickness in the prefrontal, orbitofrontal, anterior, and posterior cingulate cortices, several temporal and parietal regions, hippocampus, amygdala, thalamus, striatum, and the insula are also associated with both LLD and cognitive dysfunction. Although the callosal structure is largely preserved in LLD, WMH burden may impact its microstructure (lower fiber density, higher radial diffusivity, and lower fractional anisotropy), suggesting additional effects of vascular pathology [167].

These structural neuroimaging findings support the hypothesis that disruption of the brain networks involved in emotion regulation and connective processing by impaired structural connectivity is strongly associated with the pathophysiology of LLD [168]. The network changes mediating the relationships of WMH and subcortical lacunes with greater Geriatric Depression Scales (GDS), providing better insight into how CSVD burdens contribute to depression in both cognitively impaired and unimpaired elderly persons [74]. Several studies confirmed the importance of deep WMHs and subcortical lacunes for the risk of depressive symptoms [169,170], and a strong relationship between depression and WMH volume [171]. WMH severity was specifically correlated with decreased tissue fractional anisotropy and increased free water fraction in elderly depressed subjects, indicating that neural mechanisms in LLD may be different in patients with and without vascular damage [172].

Associations of WMHs with PSD varied according to the type of WMH and the time after stroke, such that early depressive symptoms are associated with periventricular WMHs and delayed severe depression rather with diffuse WMHs [173]. Poststroke depression (PSD) was also shown to be associated with gray matter volume loss, reduced fractional anisotropy, and increased extracellular free water (FW) in the reward system, similar to features observed in LLD without stroke [174]. One recent study reported infarcts in the right amygdala and striatum, and disconnection of right limbic and fronto–cortico–basal ganglia–thalamic circuits associated with PSD [175], while others suggested that generalized degenerative and vascular brain pathology rather than lesion-related pathology may be an important predictor for PSD [176].

While some could not demonstrate a long-term association between WMH progression and depression [177], large confluent WMHs were associated with a higher incidence of depression in individuals over age 60 years [84], with persistent depressive symptoms, poorer executive function, and cognitive impairment [22,178,179]. WMHs especially within cortico–subcortical neuronal circuits can be interpreted as sequelae of microstructural dysfunctions affecting major brain connections, indicating an association between CVD and depression [180,181], whereas, according to others, structural alterations in the cerebellum may underlie the mechanisms of depressive symptoms in patients with LLD [182].

In general, WMHs have been regarded as one of the major contributors to the vascular hypothesis of LLD and cognitive decline in the elderly, and methodologies for WMH assessment have been reviewed [72,77]. On the other hand, the cognitive reserve has long been hypothesized to provide resilience and adaptability against age- and disease-related insults. In patients with VaD, a significant interactive effect in education on the association between WMH and depression severity and language domain has been observed, suggesting that cognitive reserve, for instance, due to an interactive effect of higher education, may exert a protective effect on neurocognitive functioning in people with LLD [183].

However, not all studies supported the sole relevance of WMHs for the development of VaD [184,185]. Additional gray matter changes in the orbitofrontal cortex, medial temporal lobe/hippocampus, amygdala, and other subcortical areas, causing disruption to fronto–limbic and cortico–striatal networks, are associated with both depressive symptoms and cognitive decline [138,186]. Some studies demonstrated a distinctive association pattern between hippocampal subfield volumes and cerebral vascular burden in LLD. Structural changes in the hippocampal CA1, CA3, and dentate gyrus areas were suggested to be at the core of the underlying pathobiological mechanisms of hippocampal dysfunction in LLD [187]. Decreased anterior CBF in depressed elders may reflect decreased metabolic activity in fronto–temporal regions, while increased posterior CBF could represent compensatory processes or different activity of these networks [188].

## 11. Challenging Pathomechanisms

A growing body of evidence from neuroimaging and peripheral marker studies implicates that depressive symptoms in old age may be associated with vascular-related and other pathobiological processes, but the theory of VaD as a distinct subtype of LLD has not been fully accepted. Postmortem studies in clinically well-documented cases of LLD could not confirm the notion that diffuse WMHs, subcortical microvascular lesions, cortical microinfarcts, or AD pathology, including cerebral amyloid angiopathy (CAA), may be essential for the development of LLD [43,189,190,191,192,193], challenging the VaD hypothesis and revealing a significant gap in our understanding of the pathobiology of LLD. In this regard, it should be admitted, however, that other, non-vascular factors, such as aging, neuroinflammation [106], glial dysfunction [194,195] and amyloid pathology [144] or affection of the mesolimbic dopaminergic system [142], abnormalities in immune-inflammation response [196], impaired neuroplasticity [197], hypothalamo–pituitary–adrenal dysfunction, oxidative stress, and neurotransmitter abnormalities (e.g., serotonin, glutamate, GABA), and other hitherto unknown factors, may also contribute to VaD [168]. More recently, the role of neurotrophic factors in the pathophysiology of MDD has been discussed [198]. A GWAS provided support for the roles of vascular and inflammatory pathways in LLD. Significant enrichment of genes involved in protein degradation pathways as well as the predictive capacity of risk for cardioembolic stroke, however, support a link between LLD and risk for vascular dysfunction [199]. Other large GWAS data showed co-heritability between CRP levels and individual depressive symptoms [200] and that inflammatory markers are associated with neurovegetative symptoms of depression [201]. A GWAS of cognitive functional decline in individuals with LLD identified candidate genes, including SLC27A1, involved with processing docosahexaenoic acid (DHA), an endogenous neuroprotective component in the brain that is reduced in these patients [202]. Another study revealed that the aquapurin 4 (AQP4) locus, already known to mediate the formation of ischemic edema in the brain and heart, may play a role in the etiopathology of VaD [203]. Furthermore, genetic variants within the VCAN (versican) gene may play a role in mechanisms underlying changes of microstructural integrity of the white matter related to CVD, with an impact on AD and LLD [204]. Among all possible risk factors, CBF has received a substantial amount of attention as a distinct pathobiological factor for the development of LLD and its association with structural and functional brain changes [23]. As one important possible link between LLD and CVD, high prevalence of cardiovascular risk factors, such as hypertension, cardiac disease, diabetes, and impaired cerebrovascular reactivity in older adults, may lead to transient or permanent ischemic brain lesions, which, in turn, may result in damage of the white matter tracts mediated by demyelination, axonal degeneration, of inflammatory processes, finally causing dysfunction of brain areas and circuits engaged in emotion, behavior, and cognitive control. Postmortem stereological morphometric studies showed varying neuronal and glial pathology in depression between different age cohorts, but no clear density changes in the anterior cingulate cortex in patients with LLD [205,206].

Depression and cerebrovascular risk factors are common in late life and LLD is often present in elderly individuals with cardio- and/or cerebrovascular disease [21,22]; it is difficult to determine what is the essential causal factor and to those apart normal functioning from an abnormality in the older population. There are several possible interrelations between CVD and depressive symptoms: (1) depression is the consequence of (cerebro)vascular disease, (2) development of depression is independent of vascular disease, which, however, may stimulate the onset and course of depression, (3) CVD and depression may appear without obvious connection as two manifestations of the same genetic predisposition and pathogenetic mechanisms, (4) depression may cause/induce/promote cardiovascular and/or cerebrovascular disease, and there may be a bidirectional relationship between both disorders [207], but further studies are needed to clarify the mechanisms involved in these complex correlations. Since the temporal relationship between brain pathology and depressive and other related symptoms as well as the etiology of VaD cannot be established from post-mortem findings alone, long-term clinicopathological studies, including pre- and postmortem neuroimaging and intravitam marker studies, will be necessary to further elucidate the relations between structural/functional brain lesions, related molecular–biological cascades and depression in advanced age. Transcranial Doppler ultrasonography measuring cerebral hemodynamics may represent a valuable and inexpensive tool in the early detection, assessment, and management of VaD patients [60].

## 12. Implications for Clinical Practice and Management

LLD often affects individuals with cardio- and cerebrovascular disease and type 2 diabetes and is itself related to an increased risk to develop vascular disorders, all causing increased mortality [21,33,208]. Therefore, early recognition and treatment of all these risk factors are important, using accepted guidelines for the diagnosis and treatment of hypertension, diabetes, cardiovascular and cerebrovascular diseases, as well as early screening for LLD using adequate depression questionnaires.

Evidence-based therapies for LLD/VaD include antidepressants, electroconvulsive therapy, transcranial magnetic stimulation, and specific psychotherapies. Response to treatment is often significantly attenuated in VaD [23], so typical treatment strategies are often unsatisfactory and may not result in adequate remission of depressive symptoms [26]. VaD not only shows a poor response to antidepressive treatment and persistence of depressive symptoms but also may contribute to poor self-management of medical comorbidities [209]. A randomized controlled pharmacological trial of VaD reported a low remission rate of 33% [210]. Combined treatment with citalopram and methylphenidate demonstrated a higher rate of remission, compared with either drug alone. This treatment led to an improvement in functioning, too [211,212]. Augmentation of antidepressants with either lithium or aripiprazole was somewhat effective in LLD patients unresponsive to other antidepressants [213,214]. Regional CBF increase post-treatment was associated with decreased depressive symptoms [10]. In depressed elderly patients with cognitive impairment, a combination of antidepressants and donepezil had temporary positive effects on cognitive function but increased the risk of recurrence [215]. Fifty-nine percent of LLD patients had remitted, but less than a third of them showed cognitive improvement (29%), while this was seen in only 18% of non-remitters. Lower severity of depression, earlier onset, and greater social functioning may predict cognitive improvement with treatment for depression in elderly patients [216].

Electroconvulsive therapy (ECT) is the most efficacious treatment for LLD, with remission rates of 60–80% [217]. However, there are different notions about the impact of vascular risk factors (VRF) on the outcome of VaD therapies. Some showed superior efficacy of ECT over pharmacotherapy independent of VRFs [218], while others reported less response to ECT in patients than in those without VRFs, and the more these increased, the less antidepressive effect was observed [219]. Successful ECT was reported in a man with “probable” CAA and severe depression [220] and in a female aged 71 years with subcortical vascular encephalopathy and depression [221]. Deep transcranial magnetic stimulation was reported to be beneficial in treating VaD [222,223,224,225].

Treatment of vascular risk factors, such as hypertension improving cerebral autoregulation and leading to increased CBF, has been associated with improvement in depression [226]. Other preventive methods, including treatment of hypercholesteremia, hyperglycemia (diabetes), obesity, smoking, nutrition, lifestyle modification, or stress management, may also favorably influence the risk of CVD [227,228]. Comorbid medical disorders must be considered when planning treatment of VaD [229].

Since VaD is often associated with increased inflammation biomarkers [106], neuroinflammation may be a key therapeutic target for therapeutic strategies in LLD [67,201], and a meta-analysis suggested that nonsteroidal anti-inflammatory agents (NSAID) and cytokine antagonists have antidepressant properties in relevant patients [230]. Efficacious psychotherapies for LLD may include streamlined, neurobiologically based methods targeting behavioral domains assumed to result from dysfunctions of specific brain networks [34], or neuroplasticity-based computerized cognitive remediation programs [231], but their effectiveness needs to be validated. Cognitive behavioral therapy (CBT) may also be an efficient step towards broader treatment options for patients suffering from LLD [232].

## 13. Conclusions and Outlook

VaD as a specific subtype of LLD is a common mood disorder in aged individuals that shows a less favorable response to antidepressive therapy than MDD in younger age, in part because of the multitude of possible etiological and predisposing factors, and the lack of generally accepted diagnostic criteria. Although convincing evidence supports the role of WMHs, generalized microvascular dysfunction, vascular risk factors, such as hypertension, cardiovascular disease, or diabetes, and inflammation in the development of LLD/VaD, its essential pathomechanisms are still incompletely understood, and VaD is not included in current psychiatric diagnostic classifications. VaD has typical clinical presentations and neuroimaging findings indicating damage to cortico/fronto–striatal neuronal circuits caused by CVD. Its presenting clinical features involve poor motivation and slowed information processing as opposed to traditional mood complaint. The demonstration of deep and periventricular WMHs and subcortical gray matter lesions, as well as inflammatory biomarkers, may be helpful in the diagnosis of VaD. Preventive measures and management of VaD patients should target modifiable risk factors of vascular diseases, such as obesity, diabetes, and hypertension, that can improve vascular health as a logical approach to prevent VaD, but focused studies are needed to clarify the efficacy of such methods and their interaction with antidepressive therapies. Treatment planning should consider the modest response of VaD to antidepressants and that other methods, such as anti-inflammatory and problem-solving psychotherapies, may offer additional benefits. Since depression increases the risk of cognitive dysfunction, an early combination of antidepressants with antidementive drugs may reduce the risk of future development of dementia. However, integrated studies establishing the existence of VaD as a distinct subtype of LLD and clarifying its essential pathomechanisms and the relations of VaD to CVD and other cerebral lesions are urgently needed as a basis for early prevention and successful treatment of this frequent and devastating disorder of our aged population.

## Figures and Tables

**Table 1:** Clinical features of vascular depression (VaD) and non-VaD. Modified from [23].

Clinical Features of VaD	Clinical Features of Non-VaD
Depression occurring at age 60 to 65 years	Depression occurring at age 50 to 60 years
Absence of family history	Occasional family history
Executive dysfunctions, loss of energy, subjective feeling of sadness, apathy, anhedonia, psychomotor retardation, motivational problems, functional disability, reduced processing speed, and visuospatial skills, deficits in self-initiation, lack of insight; mild vegetative syndrome; depressive symptomatology may not meet criteria for any mood disorder requested in DSM-V	Sadness, depression according to DSM-V diagnostic criteria, marked loss of interest or pleasure in activity, changes in appetite or weight (5% of total body weight), sleep, energy, concentration, and psychomotor activity, increased suicidality, reduced verbal fluency
Higher cardiac illness burden, increased rates of vascular risk factors (hypertension, diabetes, hypercholesteremia)	Lower or same cardiac illness burden and rates of vascular risk factors (hypertension, diabetes, etc.)
Higher risk for cognitive decline and progression to cognitive impairment (dementia)	Lower or similar risk for cognitive decline and progression to dementia
Fluctuating course of cognitive impairment due to progression of white matter hyperintensities	Chronic course
Greater treatment resistance and poorer outcome	Lower or same treatment resistance and outcome (?)
Associated with increased disability and mortality	?

## Abbreviations

AD	Alzheimer disease
BBB	blood–brain barrier
CBF	cerebral blood flow
CSF	cerebrospinal fluid
CSVD	cerebral small vessel disease
CVD	cerebrovascular disease
ECT	electroconvulsive therapy
GWAS	genome-wide association study
LLD	late-life depression
LOD	late-onset depression
MCI	mild cognitive impairment
MDD	major depressive disorder
MVD	microvascular dysfunction
PSD	poststroke depression
VaD	vascular depression
VDH	vascular depression hypothesis
VEGF	vascular endothelial growth factor
VRF	vascular risk factor
WMH	white matter hyperintensities
