# Correction: Jellinger, K.A. Pathomechanisms of Vascular Depression in Older Adults. *Int. J. Mol. Sci.* 2022, *23*, 308

**DOI:** 10.3390/ijms232112949

**Published:** 2022-10-26

**Authors:** Kurt A. Jellinger

**Affiliations:** Institute of Clinical Neurobiology, 1150 Vienna, Austria; kurt.jellinger@univie.ac.at

In the original publication [1], a substantial proportion, including major parts on the role of generalized microvascular dysfunction in depression, was used from the paper by AFJ Geraets, S Köhler & MT Schram “Vascular and metabolic risk factors of late-life depression”, submitted in July 2021 to the journal *Vessel Plus* [2] without referencing this until then unpublished manuscript. The author apologizes for not having properly referenced this work. The citation has now been inserted in several paragraphs. With this correction, the order of some references has been adjusted accordingly.

The authors state that the scientific conclusions are unaffected. This correction was approved by the Academic Editor. The original publication has also been updated.
